# Foot-and-Mouth Disease in the United States

**Published:** 1881-04

**Authors:** Jas. D. Hopkins

**Affiliations:** New York City


### CORRESPONDENCE.

### FOOT-AND-MOUTH DISEASE IN THE UNIEED STATES.

Editor of Journal of Comparative Medicine:—

OWING to the apathy of our Government, and the criminal neglect of our legislators, this country has no record of the various contagious diseases which afflict our domestic animals.

The Commissioner of Agriculture at Washington has attempted to investigate the origin and spread of contagious diseases for several years past, but his efforts in this direction have been limited, owing to the non-appreciation of his actions by those who have authority to make suitable appropriations to carry such a work out successfully. Hence, we have only partial reports; but those reports show an alarming state of affairs in our country—hog cholera, contagious pleuro-pneumonia, and foot-and-mouth disease are prevalent, and no effort is made to remedy the evil. Last year, by this report, over ten millions of dollars worth of swine were lost by the plague. No estimate is made of losses by pleuro-pneumonia, and no mention is made of foot-and-mouth disease.

England, by her system of inspection of American imports of live animals, has demonstrated the fact of many contagious diseases existing among American herds.

The mortality among cattle *en route* to foreign ports is readily explained by a glance at the English reports of the condition of these animals on arrival.

Still, our country, in the face of all this accumulated evidence of the existence of contagious diseases, takes no action to check its spread, or relieve the suffering breeder by sending to his aid competent veterinarians.

I have seen foot-and-mouth disease among steers in the Central Stock Yard, Jersey City, and on the next day know that a steamship was loaded with animals from this infected yard.

Other veterinary surgeons that I have conversed with have seen footand-mouth disease at the Union Stock Yards, and still our merchants go on shipping cattle from these infected yards.

Is it any wonder that the mortality is great on shipboard, and the arrival of hundreds of sick animals are reported from foreign ports?

We have no idea of the extent of country over which foot-and-mouth disease has spread, and will not know until our Government enacts such laws that the movement of sick animals will be observed by experts, and by them traced back to their homes, and the contagion stamped out.

There is no possibility of our Government doing anything this year to stamp out, or even to control, the centre of contagion.

Yours, truly,

JAS. D. HOPKINS, D. V. S.

NEW YORK CITY, March 22, 1881.

